# Assessment of a Salt Reduction Intervention on Adult Population Salt Intake in Fiji

**DOI:** 10.3390/nu9121350

**Published:** 2017-12-12

**Authors:** Arti Pillay, Kathy Trieu, Joseph Alvin Santos, Arleen Sukhu, Jimaima Schultz, Jillian Wate, Colin Bell, Marj Moodie, Wendy Snowdon, Gary Ma, Kris Rogers, Jacqui Webster

**Affiliations:** 1 Pacific Research Centre for the Prevention of Obesity and Noncommunicable Diseases (C-POND), Fiji National University, Nasinu, Suva, Fiji; arti.pillay@fnu.ac.fj (A.P.); arleen.sukhu@fnu.ac.fj (A.S.); jillian.wate@fnu.ac.fj (J.W.); 2 The George Institute for Global Health, University of New South Wales, Sydney NSW 2052, Australia; ktrieu@georgeinstitute.org.au (K.T.); jsantos@georgeinstitute.org.au (J.A.S.); krogers@georgeinstitute.org (K.R.); 3 Independent Nutrition Consultant, Suva, Fiji; jimaima63@gmail.com; 4 Global Obesity Centre, Deakin University, Geelong VIC 3220, Australia; colin.bell@deakin.edu.au (C.B.); marj.moodie@deakin.edu.au (M.M.); wendy.snowdon@deakin.edu.au (W.S.); 5 Deakin Health Economics, Centre for Population Health Research, Faculty of Health, Deakin University, Burwood VIC 3125, Australia; 6 School of Medicine, University of Western Sydney, Campbell town, Sydney 2560, Australia; G.Ma@westernsydney.edu.au; 7 School of Public Health, University of Sydney, Sydney 2006, Australia

**Keywords:** population sodium intake, salt reduction, nutrition intervention, Pacific Islands, behavior change, health policy, salt targets, blood pressure, hypertension

## Abstract

Reducing population salt intake is a global public health priority due to the potential to save lives and reduce the burden on the healthcare system through decreased blood pressure. This implementation science research project set out to measure salt consumption patterns and to assess the impact of a complex, multi-faceted intervention to reduce population salt intake in Fiji between 2012 and 2016. The intervention combined initiatives to engage food businesses to reduce salt in foods and meals with targeted consumer behavior change programs. There were 169 participants at baseline (response rate 28.2%) and 272 at 20 months (response rate 22.4%). The mean salt intake from 24-h urine samples was estimated to be 11.7 grams per day (g/d) at baseline and 10.3 g/d after 20 months (difference: −1.4 g/day, 95% CI −3.1 to 0.3, *p* = 0.115). Sub-analysis showed a statistically significant reduction in female salt intake in the Central Division but no differential impact in relation to age or ethnicity. Whilst the low response rate means it is not possible to draw firm conclusions about these changes, the population salt intake in Fiji, at 10.3 g/day, is still twice the World Health Organization’s (WHO) recommended maximum intake. This project also assessed iodine intake levels in women of child-bearing age and found that they were within recommended guidelines. Existing policies and programs to reduce salt intake and prevent iodine deficiency need to be maintained or strengthened. Monitoring to assess changes in salt intake and to ensure that iodine levels remain adequate should be built into future surveys.

## 1. Introduction

Increased blood pressure (BP) is associated with increased risk of heart diseases and stroke. Increased dietary salt intake is associated with increased BP [1]. The World Health Organization (WHO) recommends a salt intake of less than 5 grams per day [2]. Reducing salt intake to less than 5 grams per day in adults has shown to reduce systolic blood pressure by 3.47 mmHg (0.76 to 6.20) and diastolic blood pressure by 1.81 mmHg (0.54 to 3.08) [3].

In Fiji, noncommunicable diseases (NCD) were the top ten causes of mortality in 2015 [4], with ischaemic heart diseases and hypertensive diseases accounting for 16.6% and 4.6% of proportionate mortality, respectively [4]. The 2011 WHO STEPwise approach to surveillance of noncommunicable disease risk factors (STEPS) survey showed an increase from 2002 in the prevalence of high blood pressure (systolic blood pressure (SBP) ≥ 140 and/or diastolic blood pressure (DBP) ≥ 90 mmHg or currently on medication for raised BP) from 24.2% to 31% [5]. While there are other contributors to high blood pressure, a shift in diet patterns has also been observed over time, showing more reliance on processed and imported food products [6], which tend to be higher in salt [7].

Monitoring and lowering population salt intake have been identified as important steps towards reducing the burden of NCDs [8]. In Fiji, available information for salt intake was based on 24-h recall data from the 2004 National Nutrition Survey (NNS). The estimated overall mean salt intake was 3.86 grams, which was widely understood to be an underestimation [9]. Mean data on salt intake based on 24-h urine samples was previously unavailable, which meant that targeting interventions towards specific groups and monitoring salt intakes over time was difficult. Major sources of salt in the Fijian diet, identified by the 2004 NNS 24-h recall data, were iodized table salt (27.1%), bread (17.4%), roti (14.5%), fish and seafood, including canned fish (12.1%), and meat, including canned meat (7.0%) [9]. The use of salt and salty condiments is also common in households for food preparation.

The aim of this study was to evaluate the impact of an intervention to reduce population salt intake in Fiji based on changes in mean salt intake measured through 24-h urine samples. Salt has been used as a vehicle for fortification of iodine in Fiji since 1996, in order to address iodine deficiency amongst pregnant women and school children [10]. Thus, iodine levels were also measured in this study to ensure that interventions on salt reduction did not affect iodine interventions.

The project was part of the Global Alliance for Chronic Diseases Hypertension research program.

## 2. Materials and Methods

The study took place between 2012 and 2016 in Fiji, an upper-middle income Melanesian island country in the South Pacific Ocean, north of New Zealand. Fiji has a total population of 837,271 [11], and is divided into four major jurisdictions: Central, Eastern, Western and Northern. Currently, 87% of the population live on the two major islands—with just under half of the population living in urban areas and just over half in rural areas, but with more rapid rates of population increase in urban areas. The Fiji population is categorized as iTaukei (natives of the country), Fijians of Indian descent (FID) and Fijians of Other descent (FOD) (Chinese, Pacific Islanders, Europeans, and all other nationalities) [11]. For the purposes of this study, we compared iTaukei with all other Fijians.

The study comprised three phases: (1) a nationally representative survey to measure baseline population salt consumption patterns, (2) the implementation of a multi-faceted salt reduction intervention and; (3) a survey after 20 months, to assess any changes in salt intake resulting from the intervention to date. A detailed methodology was published in the protocol [12]. Ethical Approval for the survey was granted by the University of Sydney, Human Research Ethics Council (15,359), Deakin University (2013-020), and the Fiji National Research Ethics and Review Committee (FNRERC 201307).

### 2.1. The Intervention

Between the baseline and 20-month surveys, a multi-sectorial and multi-faceted intervention targeted towards (i) food manufacturers and retailers (ii) caterers and bakers, and (iii) consumers through health workers, media and community leaders, was undertaken by the study team at the Pacific Research Centre for the Prevention of Obesity and Noncommunicable Diseases (C-POND), in collaboration with the Ministry of Health and Medical Services (MoHMS) and the National Food and Nutrition Centre (NFNC). Based on similar programs in other countries [13], and informed by the baseline monitoring of salt consumption and behaviors, the intervention aimed to increase awareness of the link between salt and health, to reduce the use of discretionary salt, and to reduce salt in the food supply through product reformulations (See Table 1). Prior to this study, salt intervention activities were implemented by the MoHMS, and voluntary salt targets for a range of food categories had been agreed by the food industry [14].

### 2.2. Participant Recruitment

Both surveys used a multi-stage cluster sampling approach to select samples representative of the adult population in Fiji. The baseline survey used the sampling frame from the WHO STEPS survey conducted in 2011. The 20-month survey used the 2014 National Nutrition Survey as the sampling frame. In both surveys, Enumeration areas (EAs) were selected through probability proportional to sampling size. Households were then randomly selected from each EA and within each household; one individual was randomly selected without replacement using the KISH method [15]. At baseline, a subsample of 600 individuals aged 25–64 years, from the total sample of 2515, was selected to participate in the salt sub-study. Based on the 40% response rate during the baseline survey, a sample of 1215 was estimated for the 20-month survey. This was to provide 80% power with an alpha of 0.05, to detect a 10% difference in salt intake from the baseline.

### 2.3. Data Collection

The objective of the surveys was to collect information on demographics and salt intake through urine samples. Data collection took place from August 2012 to the end of December 2013 at baseline, and from November 2015 to end of August 2016 at 20 months. Data collection was carried out by 60 trained Public Health nurses from their respective divisions during baseline and 8 trained research assistants carried out the survey at 20 months. The training covered recruitment, data collection, data storage and record keeping procedures. A refresher training post-cyclone Winston was conducted for the 20-month survey.

Participants were contacted via telephone and through home visits. Once the participants were recruited, and their written informed consent was obtained, they were provided with a 2.5-litre container for a 24-h urine collection, with verbal and written instructions for the collection. Participants were instructed to start the 24-h urine collection on a new day, to exclude the first urine of the day and to start the collection from the second urine till the first urine of the second day. The date, start and finish times were recorded on the bottles.

Demographic data and physical measurements (height to the nearest 0.1 cm and weight to the nearest 0.01 kg for calculating body mass index (BMI), and BP from three readings for determining blood pressure) were collected according to the WHO STEPS surveillance protocol [15], as part of the first visit. Appointments for a second visit were made to collect the 24-h urine samples. When all of the information had been collected, participants were presented with a $FJ20.00 supermarket voucher in appreciation for their participation. Urine sample volumes were measured, aliquots of 100 mL were extracted, labeled and sent to the Vanua Medical Lab where they were stored at less than 20 °C until they could be analyzed.

At 20 months, an additional spot urine aliquot for all non-pregnant females of child-bearing age between 25–45 years was sent to Westmead Hospital in Sydney for an analysis of iodine concentration.

### 2.4. Data Analysis

Analysis of the sodium and creatinine concentrations was undertaken at baseline and 20 months, with additional analysis for iodine and potassium at 20 months. Creatinine per day (mmol) was used as a marker for completion of the 24-h collection. For both baseline and the 20-month surveys, incomplete collections less than 500 mL total urine volume for both sexes, and creatinine of less than 4.0 mmol/day for women and less than 6.0 mmol/day for men were excluded [16].

Baseline and 20-month survey data were weighted by age, sex, and ethnicity (a total of 8 groups for each time point) based on the distribution of the 2007 Fiji Census. All analyses were done using the *svy command* in STATA/IC 13.1 for Windows (StataCorp LP, College Station, TX, 77845, USA), taking into account the survey design, cluster effect (EA), strata effect (division), and finite population correction factor (395,464), using the Taylor linearization approach for variance estimation [17].

## 3. Results

### 3.1. Response Rate

At baseline, of the 600 invited participants, 241 (40%) urine samples were obtained and at 20 months, of the 1215 invited participants, 497 (41%) urine samples were obtained. At baseline, 14 participants were excluded due to missing urine volumes and seven for missing demographic information. At 20 months, 58 participants were excluded for missing urine volumes and one for missing sex information. Furthermore, 51 and 166 were excluded from the analysis for suspected incomplete 24-h urine collection, leaving a total of 169 at baseline, and 272 participants at 20 months (28.2% and 22.4% participation rates).

### 3.2. Population Characteristics

Participant characteristics for both baseline and 20-month survey participants are summarized in Table 2. The mean age of the sample was 42 years for both surveys, with an almost even distribution of sex and ethnicity. For both baseline and 20-month surveys, the majority of the participants (about 80%) were from the Central and Western Divisions. About 40% were educated to primary and secondary level, while only a small proportion had no formal schooling. Aside from DBP, there were no significant differences in characteristics between baseline and 20-month survey participants (*p* > 0.05).

### 3.3. Changes in Salt Intake

The weighted mean population salt intake was 11.7 g/day (standard error (SE) 0.7) at baseline and 10.3 g/day (SE 0.5) after 20 months, but the difference was not statistically significant (−1.4 g/day, 95% confidence interval (CI) −3.1 to 0.3, *p* = 0.115). The proportion with salt intake above the WHO maximum target of 5 g/day was 86.4% and 78.2% at baseline and 20 months respectively (Table 3).

### 3.4. Subgroup Analysis Disaggregated by Gender

There was a significant reduction in salt intake among females in the Central Division from baseline to follow-up (−3.34 g/day, 95% CI −6.07 to −0.61; *p* = 0.017). In the other divisions combined, female salt intake increased although this was not statistically significant (1.23 g/day). Further analysis (i.e., difference-in-differences) showed that there was a differential effect between females in the Central Division compared to other divisions (−4.57 g/day, 95% CI −7.92 to −1.21; *p* = 0.008). There was no differential effect by division for males (0.71 g/day, 95% CI −4.64 to 6.06; *p* = 0.792). There was also no differential effect by ethnicity or age for both sexes (Table 4).

### 3.5. Comparison of Results of Those Affected and Not Affected by the Cyclone for Participants at 20 Months

Data collection in Central Division participants was completed prior to Tropical Cyclone Winston. In the regions that were affected by the cyclone, data collection was delayed for 3 months which allowed diets to return to normal. In total, 105 of the 272 participants with usable urine samples at 20 months were recruited from the affected regions, and their mean salt intake was considerably higher compared to those surveyed prior to the cyclone (Table 5).

### 3.6. Potassium and Iodine Intakes

At 20 months, mean potassium intake was 79.9 mmol/day (SE 5.0) (sodium-to-potassium ratio was 2.6 (0.1)) which is below the WHO guideline of at least 90 mmol/day for adults.

A total of 131 useable spot urine samples were collected from non-pregnant women of child-bearing age surveyed at 20 months (11% of original sample). Mean urinary iodine (UI) concentration was found to be 207 µg/L which meets the recommendations for women. However, the proportion of the sample with UI concentration less than 50 µg/L was 4.6% and the proportion of the sample with UI concentration less than 100 µg/L was 19.1%.

## 4. Discussion

This study aimed to assess the impact of a national population-wide salt reduction intervention in Fiji and highlights the need for strengthened interventions. We found that population salt intakes were high, using a 24-h urine analysis. However, we were unable to demonstrate any statistically significant reduction in population salt intake, as a result of the intervention to date. The lack of a significant effect was potentially due to the low response rate, as the sample sizes were possibly too small to detect any changes. The low response rate could also have resulted in a sampling bias which could also affect the results. The limited intervention dose, based on trying to target the whole population spread out over different islands in a relatively short timescale, might also explain the lack of impact. Whilst it is not possible to draw firm conclusions in relation to the impact on salt intake, we observed some improvements in knowledge and salt-related behaviors, suggesting that the communication campaign was starting to have an impact. These findings are being explored further in a subsequent paper.

The data does show a statistically significant reduction in salt intake for females in the Central Division, which is interesting to consider. However, the small sample sizes again mean that it is difficult to draw firm conclusions. Female salt intake was much higher at baseline in the Central Division (12.41g/day (0.83)) than in the other divisions (8.15g/day (0.71)). This could potentially be explained by the fact that there are more urban areas in the Central Division, so females are more likely to be working and have access to a greater range of processed foods and places to eat out. The higher baseline for female salt intake would make it easier to achieve a reduction. The intervention may also have been more intense in the Central Division due to its proximity to the capital city Suva, which is where the project staff and government organizations involved in the interventions are based. This data highlights the importance of disaggregating results in relation to sex, in view of the fact that females tend to have a different salt intake to males, and in order to understand any differential impact of the interventions.

The potential impact of Cyclone Winston in relation to any differential impact on the regions also needs to be considered. In contrast to other regions, the monitoring in the Central Division took place before Cyclone Winston which struck Fiji in February 2016. This means that the Central Division was the only area where people had not been provided with food rations for any period prior to the survey. Rations distributed after Cyclone Winston comprised of rice and milk, plus canned fish, canned meat, instant noodles and biscuits, most of which are contributors to dietary salt [18]. Despite the research team allowing several months after the cyclone before resuming the survey, so that people could resume their normal routines, mean salt intake amongst those surveyed after the cyclone was found to be significantly higher, 12 g/day (SE 0.7), than those surveyed before the cyclone, 9.2 g/day (SE 0.6) *p* = 0.003. Whilst it is not possible to be certain due to the small sample size, an examination of differences between the regions at baseline showed that there were no similar disparities, while the regional sub-analysis of changes in salt intake showed that there was a significant reduction in the Central Division which was not observed in the other divisions, suggesting that the cyclone may have impacted diets.

Population salt intake, measured at baseline and at 20 months, was similar to global mean estimates of population salt intake [19]. The high salt intake in Fiji could be attributed to the shift in diet patterns that has been observed over time with more reliance on processed and imported food products [6]. The link between increased salt intake and increased blood pressure, leading to cardiovascular diseases [20,21] strokes [22], and kidney diseases [23], is well established. The 2011 WHO STEPS survey showed that there had already been an increase in the prevalence of high blood pressure (SBP ≥ 140 and/or DBP ≥ 90 mmHg or people currently on medication for raised BP) from 24.2% in 2002 to 31% in 2011 [5]. There is strong evidence that reducing salt intake will reduce blood pressure, which in turn is associated with reduced risk of stroke and fatal coronary disease in adults [3].

The multi-sector, complex population-wide intervention, combining voluntary government agreements with the food industry with community mobilization and awareness in Fiji was challenging to implement effectively in the short timescale. It is unlikely that the voluntary agreements with industry were translated into significant changes in the levels of salt in the overall food supply during the intervention period. Also, limited financial and human resources mean that the reach of the communication activities may have been limited. Detailed information on different aspects of the intervention and its cost is being compiled in a separate paper focusing on the process evaluation and cost of this study. The intervention was broadly similar to the United Kingdom’s (UK) salt reduction strategy. The UK successfully demonstrated a 15% reduction in the average population salt intake during a 7-year period [16] with parallel reductions in people suffering from strokes, heart attacks, and heart failures during the same time period [16]. Multiple other countries have also effectively reduced population salt intake through similar multi-faceted interventions [24]. While this implementation science study in Fiji was unable to demonstrate a reduction in salt intake after 20 months, evidence from the UK and other countries suggests that the intervention strategies are worthwhile and should continue [24]. Adherence to agreements to reduce salt in foods by the food industry requires effective industry engagement and transparent monitoring [25,26]. Further sustained efforts will be required in Fiji, to ensure that the voluntary sodium targets that were set and accepted by the industry are adhered to in the next few years. Challenges such as costs for re-formulation and lack of control on imported products of similar categories have been highlighted as reasons for slow progress by food industries in Fiji, yet multiple other countries have overcome such challenges, demonstrating feasibility [26].

In a parallel study in Samoa, population salt intake was 7.09 g (SE 0.09) [27] which is lower than Fiji yet still higher than WHO recommendations for salt intake. Whilst there has yet to be a reduction in salt intake in Samoa [28], the Samoan Ministry of Health has integrated the regional salt targets [7] into its draft food regulations as part of a comprehensive salt reduction strategy. This is in line with growing evidence from modelling which suggests that regulated targets are more cost-effective than voluntary targets [28]. Whilst regulatory approaches need to be supplemented with behavior change interventions [29], there is increasing evidence showing that education alone is not sufficient to reduce salt intake to the recommended levels [29], especially when the majority of salt is added to foods before it is sold [30]. Whilst implementing effective policy changes might pose challenges for low and middle-income countries (LMICs) due to limited expertise and resources, there is evidence that programs can be effective [31], and reductions in salt intake have been associated with substantial cost savings [28]. Cost saving has been estimated for reduction of salt intake of 15% in LMICs with 13.8 million deaths averted over 10 years at an initial cost of less than $0.40 per person per year [30] and salt reduction is therefore listed as a top priority for action to address the non-communicable diseases crises worldwide [32].

Iodine intake has been identified as an ongoing public health concern in some Pacific Islands including Vanuatu [33] and Samoa [34]. In particular, iodine deficiency during pregnancy and lactation can negatively affect fetal development and impact health. This study confirmed that the mean iodine concentration (207 µg/L) of non-pregnant women (*n*-131) between 25–45 years was in the WHO recommended range (200–299 µg/L) based on data collected in 2016 [35]. Iodine was recognized as a public health problem in Fiji in 1996 when mean concentrations of iodine amongst school children were found to be 26 µg/L with 45% prevalence of goiter amongst pregnant women and school children [10]. Universal salt iodization, including certification of iodized salt imports was mandated that year [10]. A subsequent survey in 2009 demonstrated that the iodine status amongst children and pregnant women was now sufficient with a median of 237 µg/L and 227 µg/L, respectively. Our study provides further evidence to support the fact that Fijian adults are no longer iodine insufficient. As salt is the vehicle for iodine fortification, it is important that efforts to reduce salt are aligned with continued efforts to maintain adequate iodine intakes [35]. This requires collaboration on monitoring changes in intake and adjusting the amount of iodine added to salt accordingly [35].

### Study Strengths and Limitations

A number of strengths for the study can be noted. The study was designed to obtain a nationally representative sample of 24-h urines to assess population salt intake at baseline and after 20 months [36]. Whilst response rates were low, the comparison of the sample characteristics between the two surveys showed the samples were remarkably similar. Though 24-h urine collection can be onerous for participants and researchers, it is still regarded as a gold standard for assessing salt intake, as it accounts for around 95% of secreted sodium, although it still does not account for the possible 10% that can be lost through sweat, saliva, and gastrointestinal secretions [37]. Creatinine cutoff and total volume were used to ensure completeness of 24-h urine collections, in line with previous studies [16,38,39], so we are confident that the estimates of salt intake are fairly robust.

A weakness of the study is that the low response rates at baseline (28.2%) and at 20 months (22.5%), whilst comparable to other studies, resulted in the sample size likely being too small to measure changes in salt intake. The small sample sizes and sampling issues may have introduced biases, which may explain some of the observed findings and limit the generalizability of the findings to the Fijian population. As the study did not include a control group, it would also not have been possible to differentiate an intervention effect from secular trends. Lengthy policy change processes, which impacted intervention implementation, and the Cyclone, which delayed the planned 20-month monitoring and potentially impacted the results, were also major challenges. Restricting the intervention and monitoring to a smaller more defined geographic area—such as just the main island of Viti Levu, where the capital is based—might have been more achievable.

For future population-based implementation science studies measuring changes in salt intake, consideration should be given to having a longer, more intense intervention period and obtaining higher participant response rates for urine samples, to ensure adequate power to demonstrate an effect.

## 5. Conclusions

Whist measured salt intake was 11.7 g/day at baseline and 10.3 g/day after 20 months, it was not possible to draw firm conclusions about any possible changes due to the low response rates and high risk of sampling bias. However, this study provides credible nutrient intake data on sodium, potassium and iodine levels in Fiji through the assessment of urine samples. In order to achieve the global WHO target of reducing salt intake to less than 5 g p/d per person by 2025, existing policies and programs, which aim to change consumer behavior, reduce salt levels in foods and meals, and target schools and hospitals, need to be strengthened. Monitoring to assess population sodium and iodine levels should be built into future surveys such as the WHO STEPS survey of NCD risk factors and the National Nutrition Survey.

## Figures and Tables

**Table 1:** Intervention activities.

Strategy and Goal	Actions
Strategic health communication Goal: To increase consumer awareness on salt and health	Train and engage health educators including health workers, government workers, faith-based and voluntary organizations to disseminate messages on salt and health
	Distribution of information materials to consumers through nurses and dietitians in 21 districts Public awareness campaign on television, radio, websites, billboards “Kick the salt, lower you blood pressure”. Dissemination of communication materials: pamphlets, posters, booklets and salt message DVDs.
Industry engagement Goal: To reduce sodium content in processed foods through reformulation and improve the food environment	Engage food manufacturers to lower the salt content of foods towards the Fiji salt targets for different food categories.
	Engage food retailers to import reduced salt products and lower salt alternatives for similar brands of products. Engage restaurants, bakeries and catering facilities through training Environmental Health Officers, caterers and canteen managers on salt reduction and providing Information Education Communication (IEC) materials for dissemination. Incorporate the removal of salt shakers from tables as part of the Ministry of Health and Medical Services (MoHMS) restaurant grading scheme.
Salt reduction in the main Fiji hospital Goal: To educate and improve the food supply for hospital patients and staff	Educate hospital staff on salt and health. Educate food service staff in the preparation of low salt meals
	Improve the food environment in hospitals through removing the salt shakers from tables and lowering the sodium content of hospital meals Educate patients and their relatives on salt and health

**Table 2:** Characteristics of participants surveyed at baseline and after 20 months.

Characteristics	Unweighted			Weighted (Age, Sex, Ethnicity)			2007 Fiji Census Data (*n* = 395,464)
	Baseline (*n* = 169)	20 months (*n* = 272)	*p*-value	Baseline	20 months	*p*-value	
Age, years (mean, SE)	46.7 (0.8)	44.1 (0.6)	0.013	42.6 (0.9)	42.4 (0.6)	0.890	
Age group (%)							
25–44 years 45–64 years	42.0 58.0	53.7 46.3	0.017	63.2 36.8	63.2 36.8	1.000	63.2 36.8
Female (%)	55.6	53.7	0.690	49.1	49.1	1.000	49.1
Ethnicity (%)							
iTaukei	50.3	57.0	0.170	52.5	52.5	1.000	52.5
FID and FOD	49.7	43.0		47.5	47.5		47.5
Division (%)							
Central	42.0	40.8	0.142	42.6	40.6	0.846	40.4
Eastern ^a^	1.8	6.6		1.7	5.7		4.2
Northern	20.9	17.3		17.2	20.2		15.9
Western	37.3	35.3		38.6	35.4		39.5
Education (%)							
No formal schooling	0.0	3.0	0.014	0.0	2.4	0.299	
Primary school level	52.7	40.3		46.1	38.7		
Secondary school level	35.3	39.9		39.8	41.1		
Tertiary school level and post-graduate	12.0	16.8		14.1	17.9		
Height, cm (mean, SE)	166.4 (0.7)	167.4 (0.6)	0.310	167.9 (1.1)	167.8 (0.9)	0.924	
Weight, kg (mean, SE)	78.9 (1.5)	82.8 (1.2)	0.046	79.8 (2.1)	82.4 (1.4)	0.321	
Body Mass Index, kg/m^2^ (mean, SE)	28.4 (0.5)	29.5 (0.4)	0.072	28.2 (0.6)	29.2 (0.4)	0.199	
SBP, mmHg (mean, SE)	132.9 (1.7)	133.1 (1.2)	0.906	129.1 (1.5)	131.5 (1.3)	0.203	
DBP, mmHg (mean, SE)	82.1 (0.9)	83.4 (0.8)	0.281	80.3 (0.8)	82.8 (0.9)	0.037	
History of hypertension (%)	28.1	27.9	0.976	21.7	25.2	0.497	
Urinary volume, mL (mean, SE)	1764.3 (52.6)	1639.7 (46.0)	0.082	1788.2 (63.2)	1650.9 (61.3)	0.130	
Creatinine, mmol (mean, SE)	9.9 (0.4)	10.6 (0.3)	0.165	10.0 (0.5)	10.6 (0.3)	0.352	

FID: Fijians of Indian descent; FOD: Fijians of Other descent. ^a^ Sample size from the Eastern Division was small at both surveys (*n* = 3 at baseline; *n* = 18 at 20 months).

**Table 3:** Changes in estimated mean Fiji population salt intake (after weighting age, sex, and ethnicity).

Salt intake, g/day (mean, SE)	Baseline (*n* = 169)	20 Months (*n* = 272)	Change (95% CI)
**Overall**	11.7 (0.7)	10.3 (0.5)	−1.4 (−3.1 to 0.3); *p* = 0.115
Males	13.6 (1.2)	11.3 (0.7)	−2.3 (−5.0 to 0.4); *p* = 0.099
Females	9.7 (0.7)	9.2 (0.6)	−0.5 (−2.3 to 1.3); *p* = 0.611
**Salt intake above the WHO 5 g target (%)**	86.4	78.2	−8.2; *p* = 0.083

**Table 4:** Changes in salt intake disaggregated by gender (mean, SE) by division, ethnicity and age.

		Baseline (*n* = 169)	20 Months (*n* = 272)	Difference (95% CI, *p*-value)
**By division**				
**Central**	**Male**	13.20 (1.27)	11.28 (0.96)	−1.92 (−5.08 to 1.23); *p* = 0.230
	**Female**	12.41 (0.83)	9.07 (1.10)	−3.34 (−6.07 to −0.61); *p* = 0.017
**Other division**	**Male**	13.92 (2.02)	11.29 (0.92)	−2.63 (−6.95 to 1.69); *p* = 0.229
	**Female**	8.15 (0.71)	9.38 (0.71)	1.23 (−0.72 to 3.18); *p* = 0.213
**By ethnicity**				
**iTaukei**	**Male**	12.62 (1.13)	11.04 (0.96)	−1.58 (−4.51 to 1.34); *p* = 0.286
	**Female**	9.49 (1.00)	8.15 (0.58)	−1.34 (−3.61 to 0.91); *p* = 0.240
**FID and FOD**	**Male**	14.61 (2.05)	11.56 (0.84)	−3.05 (−7.43 to 1.32); *p* = 0.169
	**Female**	9.96 (0.75)	10.49 (1.06)	0.53 (−2.05 to 3.11); *p* = 0.683
**By age Group**				
**25–44 years**	**Male**	14.01 (1.63)	10.68 (0.87)	−3.32 (−6.97 to 0.33); *p* = 0.074
	**Female**	9.81 (0.81)	8.90 (0.66)	−0.91 (−2.93 to 1.11); *p* = 0.375
**45–64 years**	**Male**	12.82 (1.14)	12.33 (0.96)	−0.50 (−3.44 to 2.45); *p* = 0.739
	**Female**	9.55 (0.86)	9.84 (0.94)	0.29 (−2.22 to 2.81); *p* =0.817

FID: Fijians of Indian descent; FOD: Fijian of Others descent.

**Table 5:** Differences in salt intakes of people surveyed before and after the cyclone.

	Total (*n* = 272)	Affected by Cyclone (*n* = 105)	Not Affected by Cyclone (*n* = 167)	Difference (95% CI)
Salt intake, g/d (mean, SE)	10.3 (0.5)	12.0 (0.7)	9.2 (0.6)	2.8 (1.0–4.6)
